# KCTD12 modulation of GABA(B) receptor function

**DOI:** 10.1002/prp2.319

**Published:** 2017-06-07

**Authors:** Melody Li, Carol J. Milligan, Haiyan Wang, Andrew Walker, Leonid Churilov, Andrew J. Lawrence, Christopher A. Reid, Seth C. Hopkins, Steven Petrou

**Affiliations:** ^1^ The Florey Institute of Neuroscience and Mental Health Parkville Victoria Australia; ^2^ Sunovion Pharmaceuticals Inc Marlborough Massachusetts; ^3^ Department of Anatomy and Neuroscience University of Melbourne Parkville Victoria Australia; ^4^ Centre for Neural Engineering University of Melbourne Parkville Victoria Australia

**Keywords:** Baclofen, CGP7930, GABA(B)R, KCTD12

## Abstract

The molecular composition and functional diversity of native GABA_B_ receptors (GABA_B_R) are still poorly understood, thus hindering development of selective GABA_B_R ligands. Potassium channel tetramerization domain‐containing protein (KCTD) 12 is a GABA_B_R auxiliary subunit and mouse KCTD12 can alter GABA_B_R function. In this study, we sought to characterize the effects of human KCTD12 on GABA_B_R kinetics and pharmacology, using an automated electrophysiological assay. Seizure susceptibility and ethanol consumption were also investigated in a KCTD12 knockout mouse model. Human KCTD12 co‐expression altered the kinetics of GABA_B_R‐mediated GIRK channels, speeding rates of both activation and desensitization. Analysis of concentration‐response curves showed that KCTD12 coexpression did not alter effects of the agonists GABA or baclofen on GABA_B_R. KCTD12 coexpression enhanced the potentiating effects of the positive allosteric modulator CGP7930, and its effects on GABA_B_R activation and desensitization. The function of KCTD12 in vivo was examined, using the KCTD12 knockout mouse model. The knockout mice were more resistant to a pentylenetetrazole proconvulsant challenge suggesting reduced seizure susceptibility. In the two bottle preference test, KCTD12 knockout mice demonstrated a reduced consumption at high ethanol concentrations. In summary, human KCTD12 accelerated the kinetics of GABA_B_R in vitro, in a manner possibly sensitive to allosteric pharmacological modulation. This study also provides novel in vivo evidence that the interaction between KCTD12 and GABA_B_R is of physiological significance, and may be a mechanism to more selectively modulate GABA_B_R.

**Abbreviations**
CGP7930: 2,6‐ditert‐butyl‐4‐(3‐hydroxy‐2,2‐dimethyl‐propyl)‐phenol
GABA*γ*: aminobutyric acid
GIRK: G protein‐activated inward rectifying potassium channels
KCTD12: potassium channel tetramerization domain‐ containing proteins12

## Introduction

GABA_B_ receptors (GABA_B_R) are heterodimeric G protein–coupled receptors consisting of the GABA_B(1a/b)_ and GABA_B(2)_ subunits. Upon GABA_B_R activation, G_*α*i/*α*o_ and G_*βγ*_ proteins are liberated and mediate postsynaptic slow inhibition via G protein‐activated inward rectifying potassium channels (GIRK, also known as Kir). The liberated G_*βγ*_ proteins also reduce presynaptic transmitter release by inhibiting voltage‐gated calcium channels. GABA_B_Rs are implicated in a range of diseases and are drug targets for multiple therapeutic indications, including muscle spasticity, seizure, and drug addiction (Bettler et al. 2004). Baclofen is used clinically and is a direct GABA_B_R agonist. However, due to the nonselective activation of GABA_B_R, its use is often limited by side effects that can include sedation and motor impairment (Hering‐Hanit 1999; Lind et al. 2004). The scope for discovery of selective GABA_B_R ligands is limited because of the lack of molecular diversity of GABA_B_R subunit isoforms, and the overlapping pharmacological profiles of the two main receptor combinations (Brauner‐Osborne and Krogsgaard‐Larsen 1999; Green et al. 2000). Despite this apparent lack of molecular diversity, the functional repertoire of native GABA_B_Rs is quite broad and varies across different brain regions, suggesting the existence of modulators of GABA_B_R function (Bonanno and Raiteri 1993; Cruz et al. 2004; Hayasaki et al. 2012; Hensler et al. 2012).

GABA_B_R can interact with other proteins such as the scaffolding proteins Tamalin and MUPP1 (Kitano et al. 2002; Balasubramanian et al. 2007) and these associated proteins are believed to underlie the functional diversity of GABA_B_R and provide potential novel mechanisms to modulate GABA_B_R (Lujan and Ciruela 2012). Proteomic analysis in rodent brains identified GABA_B_R auxiliary subunits belonging to the potassium channel tetramerization domain‐ containing protein (KCTD) family. The KCTD proteins identified were KCTD8, 12 and its isoform 12b, and 16 (Bartoi et al. 2010; Schwenk et al. 2010). These KCTD proteins are characterized by the presence of a conserved T1 domain of the voltage gated potassium channels (Stogios et al. 2005), followed by H1 and H2 domains in the carboxy‐terminal for KCTD8 and 16, while KCTD12 lacks the H2 domain (Seddik et al. 2012). Functional studies, using mouse KCTD proteins showed that all KCTDs increased activation rate of the GABA_B_R response, but only KCTD12 increased desensitization of receptor response (Schwenk et al. 2010; Seddik et al. 2012; Ivankova et al. 2013; Turecek et al. 2014). The GABA_B_R response desensitization caused by KCTD12 has been attributed to the H1 domain, whereas the H2 domain was shown to inhibit the effect of H1 domain in KCTD8 and 16 (Seddik et al. 2012). Further studies revealed that KCTD12 mediates the desensitization of GABA_B_R response by interacting with the liberated G_*βγ*_ subunit upon GABA_B_R activation (Turecek et al. 2014). In addition to altering the kinetics of GABA_B_R responses, mouse KCTD12 has been shown to increase both GABA_B_R agonist potency and plasma membrane expression (Bartoi et al. 2010; Schwenk et al. 2010; Ivankova et al. 2013).

A single‐nucleotide polymorphism in the gene promoter region of the human *KCTD12* gene was associated with Bipolar I disorder, a GABA_B_R implicated disease (Fatemi et al. 2011; Lee et al. 2011; Sand et al. 2012). The potential role of KCTD12 in neuropsychiatric disorders is further supported by altered response to fear conditioning in KCTD12 knockout mice (Cathomas et al. 2015). However, other GABA_B_R implicated diseases such as altered brain excitability and alcohol abuse have not been explored.

The effects of KCTD12 on the GABA_B_R kinetics and pharmacology would represent a unique opportunity to discover novel and more selective ways to modulate GABA_B_R. Earlier work characterized function using mouse proteins (Schwenk et al. 2010; Seddik et al. 2012; Ivankova et al. 2013; Turecek et al. 2014). Here, we sought to examine the effects of human KCTD12 on GABA_B_R kinetics, pharmacology of GABA_B_R ligands, and to explore the influence of KCTD12 on GABA_B_R function in two tests of disorders related to seizure and ethanol intake in the KCTD12 knockout mouse model.

## Materials and Methods

### Electrophysiology recording in oocytes

Stage V or VI oocytes were surgically removed from *Xenopus laevis* and were prepared as described previously (Petrou et al. 1997). Oocytes were kept in ND96 solution and stored at 16°C. Human cDNAs *GABBR1B* (NM_021903), *GABBR2* (NM_005458), *GIRK1* (NM_002239), *GIRK2* (NM_002240), and *KCTD12* (NM_138444.3) were synthesized by Genscript (Piscataway, NJ), and were subcloned into an oocyte high expression vector (Liman et al. 1992). *KCTD12* was codon optimized for oocyte expression. cDNA's were transcribed in vitro (mMessage mMachine, Ambion, Austin, TX), and 40 nl of capped cRNA was injected into each oocyte by the Roboocyte version 1 (Multi Channel Systems, Reutlingen, Germany). A total of 4–19 ng of cRNA was injected into each oocyte. The ratio of the cRNA mixture was 1:1:1:1:15 for GIRK1:GIRK2:GABBR1B:GABBR2:KCTD12. The cRNA ratio was chosen based on the amount of human KCTD12 required to observe a similar level of relative desensitization, a key signature of KCTD12, in a previous study (Schwenk et al. 2010) as shown in Figure S1. After 2–3 days, two electrode voltage clamp recording was performed, using the Roboocyte version 1. Before recording, the oocytes were placed in the bath solution that contained (in mmol·L^−1^) 52 NaCl, 40 KCl, 1.8 CaCl_2_, 1 MgCl_2_ and 5 HEPES, pH 7.4, for a minimum of 20 min to allow basal GIRK current rundown to stabilize (Vorobiov et al. 1998). Oocytes were impaled with electrodes that contained 1.5 mol·L^−1^ K‐acetate and 0.5 mol·L^−1^ KCl and were held at −50 mV. All drug application times were 60 sec followed by 6 min wash out. Recording frequency was 100 Hz and temperature was maintained between 20 and 22**°**C.

### KCTD knockout mouse model

The KCTD12 knockout mouse model was kindly provided by Professor Bettler and Dr. Gassmann from the University of Basel (Turecek et al. 2014). All studies involving the mouse model were carried out in accordance with the Guide for the Care and Use of Laboratory Animals and were approved by the Florey Institute Animal Ethics Committee. The KCTD12 knockout mouse model is on a C57/Bl6J background. All animals were maintained in a temperature controlled room, with a 12 h light on/off cycle and free access to food and liquid. Experimenter was blinded to the genotypes in the behavioral studies.

### Pentylenetetrazole‐induced seizure model

P40‐45 mice were injected subcutaneously with 100 mg·kg^−1^ of pentylenetetrazole (PTZ). Mice were placed in a clear chamber immediately after injection and the time to tonic hind limb extension was recorded.

### Two bottle preference test

Adult male mice (approximately 9 weeks old) were habituated to the holding room environment. The mice were single housed with access to two bottles of water ad libitum. A week later, one bottle of the water was replaced with 5% (v v^−1^) ethanol, thus mice were given a choice of ethanol or water. The concentration of ethanol was increased in 5% increments every 2 weeks, up to 20% (Moore et al. 2007). The position of the bottles was changed randomly to prevent side preference. Fluid in bottles was replenished weekly. Bottles were weighed to indicate the fluid consumed from each bottle.

### Drugs

GABA and baclofen (Sigma, St. Louis, MO) were prepared in bath solution just prior to experimentation. CGP7930 (Tocris, Bristol, U.K.) was dissolved in DMSO at 10 mmol·L^−1^ concentration and stored at −20**°**C until use. Pentylenetetrazole (Sigma, St. Louis, MO) was dissolved in 0.9% saline just prior experiments. Ethanol (LabServ, Australia) was diluted with water to desired concentration on a weekly basis.

### Data analysis

Electrophysiological data were analyzed, using AxoGraph (AxoGraph Scientific, Sydney, Australia). Changes in GABA_B_R kinetics were quantified with two parameters: the 20–80% rise time and relative desensitization, which was calculated as 100*(1−(end of agonist application response/maximum agonist response)) (Schwenk et al. 2010). Data from electrophysiology and PTZ‐induced seizure model are presented as mean ± S.E.M, and statistical analysis was performed on Graph‐Pad Prism (GraphPad Software, La Jolla, CA). Data from two bottle preference tests are presented as mean ± 95% confidence interval (95% CI) and random‐effect generalized least‐square regression models were performed, using Stata (StatCorp LP, College Station, TX).

## Results

### Human KCTD12 co‐expression altered GABA_B_R‐activated GIRK kinetics

**Figure 1:**
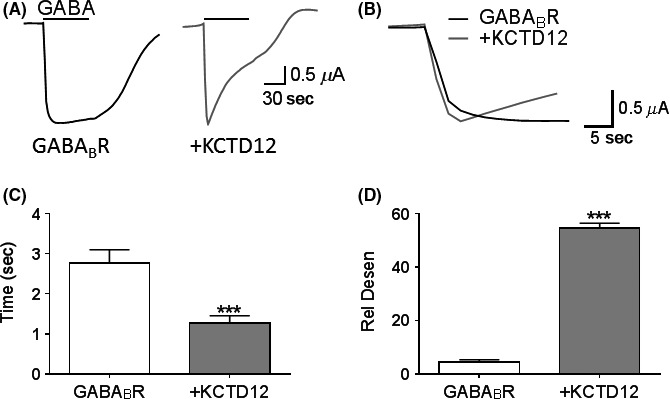
Effect of human KCTD12 on GABA_B_R kinetics. (A) Representative current traces from oocytes expressing human GABA_B_R or co‐expressing human KCTD12 (+KCTD12) in response to 1 min application of 100 *μ*mol·L^−1^ GABA. Scale bar applies to both traces. (B) Close up of the rising phase of the GABA‐activated response in Fig. 1A. (C) 20–80% rise time of GABA_B_R (*n* = 12) and +KCTD12 (*n* = 10). (D) Relative desensitization of GABA_B_R (*n* = 12) and +KCTD12 (*n* = 13). Unpaired *t*‐test, ***P <* *0.001

A single concentration of GABA (100 *μ*mol·L^−1^) was applied for 60 sec onto oocytes expressing GABA_B_R with or without co‐expressed human KCTD12 subunits (Fig. 1A). Compared to GABA_B_R only expressing oocytes, KCTD12 co‐expression significantly reduced the 20–80% rise time by around two‐fold (*P *<* *0.001) (Fig. 1B). KCTD12 co‐expression also significantly increased the relative desensitization to 54.7 ± 1.8%, as compared to 4.54 ± 0.80% in oocytes expressing only GABA_B_R (*P *<* *0.001) (Fig. 1C). Therefore, co‐expression of human KCTD12 altered the activation and desensitization kinetics of GABA_B_R in similar manner as described with mouse KCTD12 (Schwenk et al. 2010; Seddik et al. 2012; Ivankova et al. 2013; Turecek et al. 2014).

### Effects of human KCTD12 on GABA_B_R agonist pharmacology

**Figure 2:**
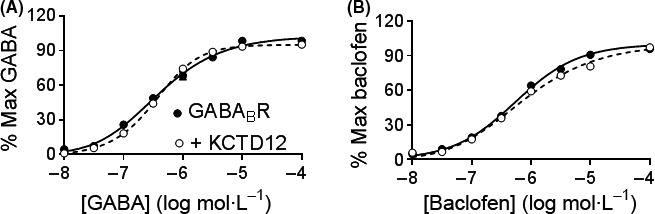
Human KCTD12 did not alter GABA_B_R sensitivity to agonists. Concentration response curves of (A) GABA and (B) baclofen in the presence and absence of human KCTD12. Responses were normalized to the maximum response in each oocyte. *N* = 5–25 at each agonist concentration.

Based on altered response kinetics, we next tested whether agonist potencies, GABA or baclofen, were changed by human KCTD12 co‐expression. Concentration‐response curves were constructed by applying 100 *μ*mol·L^−1^ agonist followed by washout and an agonist concentration ranging from 10 nmol·L^−1^ to 100 *μ*mol·L^−1^. Agonist concentrations could not be applied cumulatively due to KCTD12‐induced desensitization. Concentration response to agonists was normalized to the 100 *μ*mol·L^−1^ response obtained in each oocyte and sigmoidal concentration‐response curves were fit by nonlinear regression. Concentration response curves for both GABA and baclofen were similar between oocytes with or without co‐expressed KCTD12 (Fig. 2). The logEC_50_ for GABA was −6.43 ± 0.1 in oocytes expressing GABA_B_R alone and −6.46 ± 0.03 with KCTD12 co‐expression. The logEC_50_ for baclofen was −6.26 ± 0.02 for GABA_B_R‐expressing oocytes and −6.16 ± 0.04 for KCTD12 co‐expression.

### Effects of human KCTD12 on CGP7930 pharmacology

**Figure 3:**
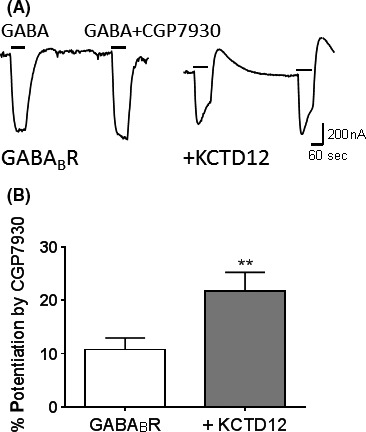
The positive allosteric modulation of CGP7930 is enhanced by KCTD12. (A) Representative current traces from oocytes expressing human GABA_B_R or co‐expressing human KCTD12 (+KCTD12) in response to 1 min application of EC_20_ GABA (100 nmol·L^−1^) followed by washout and then 1 min application of EC_20_ GABA and 10 *μ*mol·L^−1^ of CGP7930. Scale bar applied to all traces. (B) The positive allosteric modulator CGP7930 potentiation of EC_20_ GABA of oocytes expressing GABA_B_R (*n* = 36) and +KCTD12 (*n* = 28), unpaired *t*‐test, **P <* *0.01

KCTD12 is believed to interact with the R2 subunit of GABA_B_R (Bartoi et al. 2010; Correale et al. 2013). The R2 subunit is thought to contain the binding site of the GABA_B_R‐positive allosteric modulator, CGP7930 (Binet et al. 2004). Therefore, we first examined whether the presence of KCTD12 affects the potentiation of EC_20_GABA by CGP7930. Positive allosteric modulation by CGP7930 (10 *μ*mol·L^−1^) was determined at the EC_20_ concentration of GABA (100 nmol·L^−1^) (Fig. 3A). When GABA_B_R was expressed alone, the average current amplitude before and after CGP7930 were 493 ± 0.0234 nA and 544 ± 0.0279 nA, respectively, thus CGP7930 potentiated EC_20_GABA by 10.8 ± 2.15%. When KCTD12 was co‐expressed, average current amplitude before and after CGP7930 were 471 ± 0.0281 nA and 572 ± 0.0367 nA, respectively. CGP7930 potentiation was significantly increased to 21.7 ± 3.55% with KCTD12 co‐expression (*P *<* *0.01, Fig. 3B).

**Figure 4:**
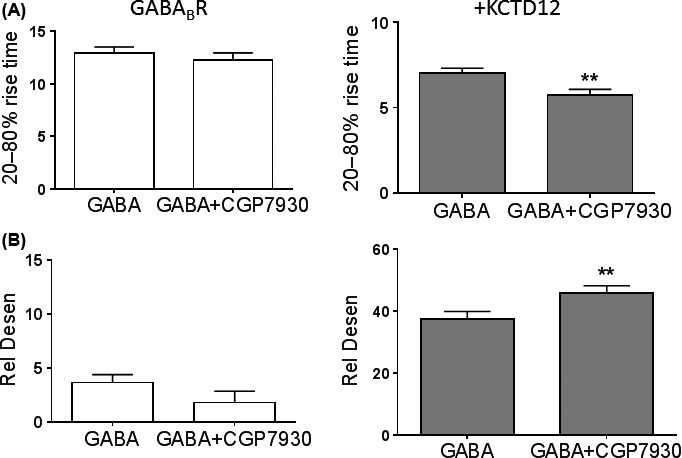
CGP7930 further accentuates the effect of KCTD12 on GABA_B_R kinetics. Effect of CGP7930 on (A) GABA_B_R 20–80% rise time and (B) relative desensitization for GABA_B_R expression only (*n* = 24) or with KCTD12 co‐expression (*n* = 18). Paired *t*‐test, **P <* *0.01

We next examined whether CGP7930 altered the effects of KCTD12 co‐expression on GABA_B_R kinetics. In oocytes that only expressed GABA_B_R, CGP7930 did not affect the 20–80% rise time or relative desensitization in oocytes that only expressed GABA_B_R was not altered by CGP7930 (Fig. 4). In KCTD12‐expressing oocytes, CGP7930 had an effect on receptor kinetics, where it shortened the 20–80% rise time by 18.4% and further accelerated desensitization by 22.5% (*P *<* *0.01, Fig. 4).

### Reduced seizure susceptibility in KCTD12 knockout mice

**Figure 5:**
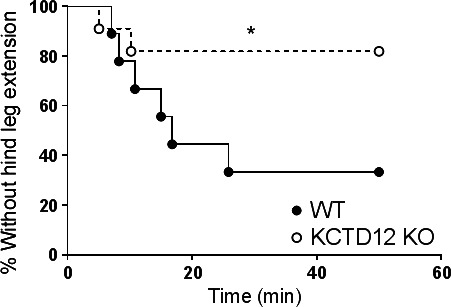
Susceptibility to PTZ‐induced seizure in the KCTD12 knockout mouse model. Survival curves of wild type (WT,* n* = 9) and KCTD12 knockout mice (KCTD12 KO,* n* = 11) to subcutaneous PTZ (100 mg·kg^−1^) induced seizure. Mantel‐Cox test, *P <* *0.05

Since KCTD12 co‐expression profoundly accelerated the desensitization of GABA_B_R responses in vitro, we hypothesized that deleting KCTD12 expression would increase the amount of inhibition mediated by GABA_B_R in vivo. Seizure is a result of brain hyper‐excitability and a decrease in seizure susceptibility would indicate higher general inhibition in brain. The proconvulsant, PTZ (100 mg·kg^−1^) was injected into wild type and KCTD12 knockout mice, and the time to seizure, as indicated by hind leg extension, was measured. For wild type, 6 out of 9 mice showed hind leg extension after 30 min of PTZ treatment (Fig. 5). For KCTD12 knockout mice, only 2 out of 11 mice showed hind leg extension, the rest of the group did not enter into the seizure state. KCTD12 knockout mice were less susceptible to chemically induced seizure (*P *<* *0.05), suggesting that GABA_B_R mediated inhibition was stronger in the KCTD12 knockout mouse model in comparison with the wild‐type mice.

### KCTD12 modulates ethanol intake

**Figure 6:**
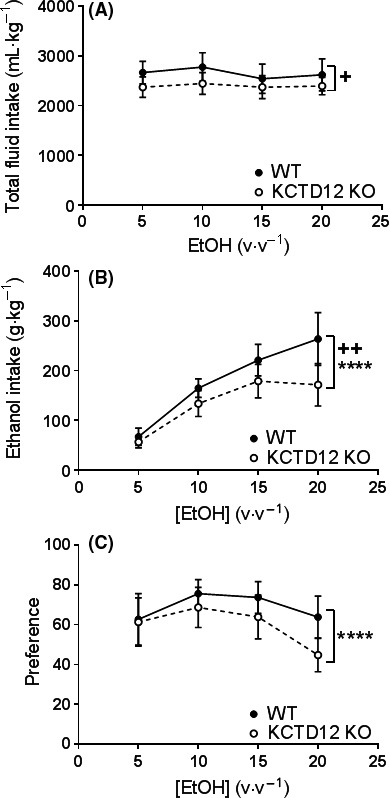
Ethanol preference test in the KCTD12 knockout mouse model. (A) Total fluid intake (water and ethanol containing water) for wild type (*n* = 9) and KCTD12 knockout mice (*n* = 10). Fluid intake was stratified by the concentration of ethanol. (B) The amount of ethanol consumed. (C) Preference for ethanol as indicated by the percentage of ethanol consumed. Note that data is presented as mean ± 95% CI interval. Random‐effect generalized least‐square test, +*P *<* *0.05, ++*P *<* *0.005 for significant effect of genotype, *P <* *0.05 for significant interaction.

To further explore the hypothesis that deleting KCTD12 expression would increase the amount of inhibition mediated by GABA_B_R in vivo, we tested another GABA_B_R‐related behavior, voluntary ethanol intake. Increased GABA_B_R function decreases ethanol intake and self‐administration in animal studies (Colombo et al. 2000, 2006; Besheer et al. 2004; Liang et al. 2006), and several clinical studies have suggested baclofen as a treatment for alcohol dependence (Addolorato et al. 2002, 2007; Morley et al. 2014). In this study, alcohol intake was assayed using two bottle preference test. Random‐effect generalized least‐square regression models were used due to this method's ability to examine the association between the genotype and the parameters measured (total fluid intake, ethanol intake and preference) at different ethanol concentrations, as well as examining the interaction by genotype and ethanol concentration. Furthermore, appropriate effect size estimates and 95% confidence interval can be reported in addition to *P* values. During the 8 weeks of experimentation, the average total fluid intake (TFI) of wild type was 127.39 mL·kg^−1^ higher than the KCTD12 knockout mice (95% CI: 6.16–248.62, *P *=* *0.04) (Fig. 6A). However, no statistically significant interaction was found between genotype and ethanol concentration (*P *=* *0.43) on TFI. Next the amount of ethanol intake was examined and we observed that during 8 weeks of experimentation, wild type mice consumed on average 21.94 g∙kg^−1^ more ethanol than the KCTD12 knockout mice (95% CI: 6.85–37.03, *P *=* *0.004) (Fig. 6B). Moreover, an interaction was found between amount of ethanol consumed and ethanol concentration (*P *<* *0.0001), indicating that the difference in amount of ethanol consumed between genotypes increased as ethanol concentration increased. Post hoc analysis revealed that the average differences in the amount of ethanol consumed between genotypes were 5.16 g∙kg^−1^ (95% CI: −3.66–14.0) at 5% ethanol, 15.57 g∙kg^−1^ (95% CI: 1.45–29.7) at 10% ethanol, 21.00 g·kg^−1^ (95% CI: 0.97–40.94) at 15% ethanol and 46.08 g·kg^−1^ (95% CI: 17.11–75.04) at 20% ethanol. To determine whether the wild type simply consumed more fluid than mutants or whether the effect was specific for ethanol, the preference for ethanol, calculated as the percentage of ethanol intake over TFI, was examined. After adjustment for TFI, on average the genotype had no effect on ethanol preference. However, a statistically significant interaction was found between preference and ethanol concentration (*P *<* *0.0001) (Fig. 6C). Consistent with the analysis on amount of ethanol consumed, the difference in ethanol preference between genotypes increased as ethanol concentration increased. Post hoc analysis showed that the average differences in ethanol preference between genotypes were −1.06 (95% CI: −15.63–13.5) at 5% ethanol, 6.32 (95% CI: −2.93–15.58) at 10% ethanol, 10.45 (95% CI: 1.15–19.75) at 15% ethanol and 18.02 (95% CI: 8.31–27.73) at 20% ethanol.

## Discussion

GABA_B_R‐mediated inhibition of CNS excitability presents a therapeutic opportunity in a variety of diseases, but lack of specific pharmacology has potentially limited its application (Bonanno and Raiteri 1993; Cruz et al. 2004; Hayasaki et al. 2012). Recapitulation of native‐state conditions in assays may improve the ability to achieve more selective compounds and here we explored the idea that the interaction between KCTD12 and GABA_B_R may be a more specific way to affect GABA_B_R for therapeutic benefit. We first developed an electrophysiological assay which showed that the human form of KCTD12 shortened GABA_B_R responses and enhanced the positive allosteric modulation of CGP7930. Using the KCTD12 knockout mouse model, we showed that deleting KCTD12 expression reduced seizure susceptibility and decreased preference for higher ethanol concentration.

Previous in vitro analysis of KCTD12 used the mouse isoforms (Schwenk et al. 2010; Seddik et al. 2012; Ivankova et al. 2013; Turecek et al. 2014; Rajalu et al. 2015). Here, we report on the ability of human KCTD12 to alter GABA_B_R kinetics. Similar to the mouse isoform, human KCTD12 accelerated rise time and desensitization of GABA_B_R. Moreover, the magnitude of modulation by KCTD12 was similar between the mouse and human isoforms (Schwenk et al. 2010). We examined two GABA_B_R agonists, GABA and baclofen, and found that their potencies were not affected by KCTD12 co‐expression. These results are in contrast to a study that showed co‐expression of mouse KCTD12 increased baclofen potency on GABA_B_R‐activated calcium channel currents in CHO cells (Schwenk et al. 2010). A recent binding assay study proposed that mouse KCTD12 increases GABA_B_R agonist potency via effects on G‐protein signaling, instead of affinity (Rajalu et al. 2015). Since there are known differences between endogenous mammalian and oocyte G‐protein signalling, differences between the baclofen concentration‐response curves in the earlier study and in present study may be due to the different heterologous expression systems used. However, in this study, expression of human KCTD12 altered the response of GABA_B_R's to CGP7930 by enhancing the extent of positive modulation and also by accelerating activation and inactivation kinetics. A recent study using mouse KCTD12 and a different GABA_B_R positive allosteric modulator GS39783 also observed the accelerated GABA_B_R kinetics, yet GS39783 positive allosteric modulation was not enhanced by mouse KCTD12 co‐expression (Rajalu et al. 2015). The mechanism underlying this discrepancy is unclear but could be due to the potential differences in species, measuring methods and heterologous expression system used.

Proteomic analysis showed that KCTD12 was bound to GABA_B_R via a constitutive interaction with G*βγ* protein (Turecek et al. 2014). Upon GABA_B_R activation, KCTD12 is believed to mediate desensitization by directly binding liberated G*βγ* proteins thereby interfering with G*βγ* protein binding and activation of GIRK channels. This activity‐dependent binding of KCTD12 to G*βγ* protein was termed dynamic binding (Turecek et al. 2014). One interpretation of our CGP7930 result is that CGP7930 binding of GABA_B_R reduces the amount of constitutively bound KCTD12 and that enhanced currents are due to the consequent greater initial liberation of non‐KCTD12 bound G*βγ* protein. This is followed by a greater amount of dynamic binding caused by higher levels of free KCTD12. This scenario could explain the greater extent of modulation and the faster activation and deactivation kinetics in the presence of CGP7930 during activation GABA_B_R in the presence of KCTD12.

Despite our increasing knowledge of the details of KCTD12 signaling, the physiological relevance of KCTD12 remains elusive. The KCTD12 homozygous knockout mice displays increased fear learning to conditioned stimulus (Cathomas et al. 2015). Increased fear learning was also observed in rats treated with baclofen (Heaney et al. 2012), suggesting that KCTD12 deletion has a similar effect to increased GABA_B_R function. Pharmacological enhancement of GABA_B_R function in seizures (Mares 2012) and ethanol intake (Colombo et al. 2000, 2006; Addolorato et al. 2002, 2007; Besheer et al. 2004; Liang et al. 2006). Here, we specifically examined the role of KCTD12 in the PTZ‐induced seizures and voluntary ethanol consumption. Our studies showed that KCTD12 knockout mice were protected from PTZ‐induced seizures and showed reduced preference for high concentrations of ethanol compared to wild types. This is consistent with the idea that a lack of KCTD12 is enhancing GABA_B_R function in the knockout mouse.

In summary, our study showed that the co‐expression of human KCTD12 shortened GABA_B_R responses. We also found that although the expression of human KCTD12 subunit did not alter the potency of GABA_B_R agonists, it did increase the allosteric modulation of GABA_B_R by CGP7930. in vivo studies in the KCTD12 knockout mice confirmed the in vitro notion that deletion of KCTD12 resembles GABA_B_R enhancement, demonstrating a potential novel target for regional and specific modulation of GABA_B_R function.

## Author Contributions

Participated in research design: All authors. Conducted experiments: Li, Reid. Performed data analysis: Li, Walker, Churilov, Lawrence, Reid, Petrou. Wrote or contributed to the writing of the manuscript: All authors.

## Disclosure

None declared.

## Supporting information


**Figure S1.** Dose‐dependent effect of human KCTD12 on GABA_B_R response desensitization.
