# Supplementary figures and images for: KCTD12 modulation of GABA(B) receptor function
Source: Pharmacol Res Perspect. 2017 Jun 7;5(4):e00319. doi: 10.1002/prp2.319 (PMC5508304; doi:10.1002/prp2.319)

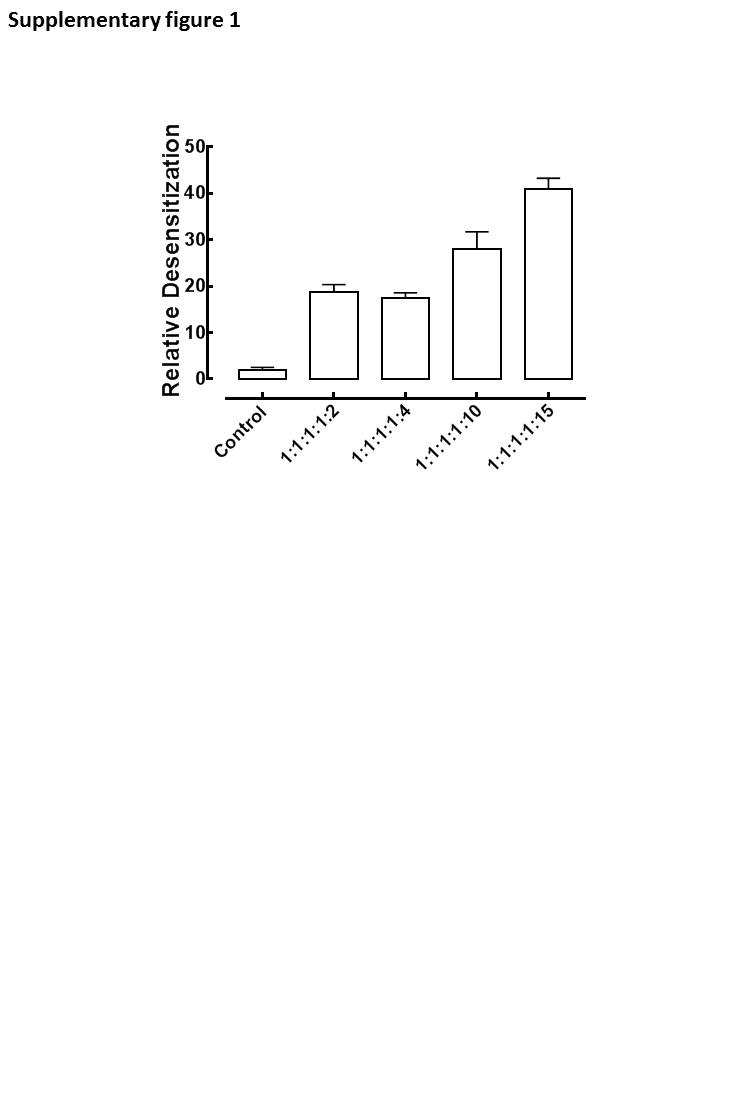

Supplement: Supplementary file 1 — Figure S1. Dose‐dependent effect of human KCTD12 on GABABR response desensitization. [file PRP2-5-e00319-s001.tif]
